# Nocturnal Oxygen for High Altitude Travel in Patients With Chronic Obstructive Pulmonary Disease

**DOI:** 10.1001/jamanetworkopen.2020.8022

**Published:** 2020-06-01

**Authors:** Daniel Combs, Sairam Parthasarathy

**Affiliations:** Department of Pediatrics, Division of Pulmonary and Sleep Medicine, University of Arizona, Tucson; University of Arizona Health Sciences Center for Sleep & Circadian Sciences, Division of Pulmonary, Allergy, Critical Care and Sleep Medicine, University of Arizona, Tucson; University of Arizona Health Sciences Center for Sleep & Circadian Sciences, Division of Pulmonary, Allergy, Critical Care and Sleep Medicine, University of Arizona, Tucson

An estimated 384 million individuals worldwide have chronic obstructive pulmonary disease (COPD), and it ranks as the third most common cause of death.^1^ Travel to high altitude, as well as travel via commercial aircraft, is common in modern society, and a substantial portion of travelers are individuals with COPD. Current guidance suggests that the use of supplemental oxygen is not needed for travel to high altitude for individuals with mild-to-moderate COPD^2^ and that oxygen is only recommended for air travel for individuals with very severe COPD.^3^

The study by Tan and colleagues^4^ has provided new support for the potential use of oxygen for individuals with COPD who are not currently considered to need supplemental oxygen at high altitude. In their placebo-controlled, randomized clinical trial evaluating the effects of nocturnal oxygen therapy, they found that nocturnal oxygen therapy significantly improved both nocturnal hypoxemia and altitude-associated periodic breathing in individuals with moderate-to-severe COPD newly arrived from 490 m (1607 ft) to 2048 m (6719 ft) above sea level. In addition to the improvement of nocturnal hypoxemia and sleep-disordered breathing seen with nocturnal oxygen therapy, they found improvement across a range of secondary outcomes, including improved objective and subjective sleep quality. The authors also reported that nocturnal oxygen therapy was associated with a decreased incidence of altitude-related adverse health events, a composite measure of hypoxemia (<75% oxygen saturation for >30 minutes), COPD exacerbations, and unstable cardiovascular disease. They found an altitude-associated adverse health event incidence of 26% during placebo nights compared with 4% during nights when receiving nocturnal oxygen therapy.

Although the findings related to sleep-disordered breathing and nocturnal oxygen saturation were significant, Tan et al^4^ did not find statistically significant differences in other relevant functional measures. Specifically, there were no differences in 6-minute walk distance, perceived dyspnea, pulmonary function test, or psychomotor vigilance test results. It should be noted that these were secondary outcomes that the study was not powered to evaluate, and a larger sample size may have found significant differences in these outcome measures.

The most striking findings from the study by Tan et al^4^ are the surprisingly severe effects of a modestly high altitude on individuals with less severe COPD who are not typically evaluated for supplemental oxygen need, which appear to be mitigated by the use of nocturnal oxygen therapy. The study included individuals with Global Initiative for Obstructive Lung Disease stages 2 to 3, with nearly three-quarters (72%) of individuals having only moderate COPD (Global Initiative for Obstructive Lung Disease stage 2). Cardiovascular disease is common in individuals with COPD, and worsening pulmonary status is associated with an increased risk of death from cardiovascular disease in patients with COPD.^5^ Altitude-induced hypoxemia in individuals with COPD can conceivably exacerbate cardiovascular events. In the study by Tan et al,^4^ 7 participants were randomized to placebo first with no cardiovascular events, and another 9 participants were randomized to placebo first with 2 withdrawn because of cardiovascular events, which yields a total of 12.5% of participants who were randomized to placebo first and had to be withdrawn from the study compared with no participants who were randomized to nocturnal oxygen therapy first. This raises concern that individuals with moderate-to-severe COPD may have serious health risks associated with travel to high altitude. Fortunately, nocturnal oxygen therapy was effective in reducing hypoxemia, and unstable cardiovascular disease was not seen in participants receiving nocturnal oxygen therapy.

The altitude exposure used in this study was only 2048 m (6719 ft) above sea level and is comparable to the typical cabin pressure used in commercial flights (2438 m [8000 ft]). Current guidelines recommend evaluation for supplemental oxygen during flights only for patients with very severe COPD (forced expiratory volume in 1 second <30% of predicted).^3^ This study by Tan et al^4^ revealed significant altitude-induced hypoxemia in individuals with lung function (forced expiratory volume in 1 second) well above this current recommendation. Given that respiratory events are among the most common medical emergencies on flights,^6^ this study suggests that a more rigorous preflight evaluation, such as hypoxia altitude simulation testing,^7^ should be undertaken in patients with COPD. This may be particularly relevant for intercontinental flights, which are often longer than 10 hours and during which travelers are likely to sleep, potentially further worsening hypoxemia during sleep.

Tan et al^4^ should be commended for a rigorous study of the potential adverse effects of high-altitude travel, as well as offering a solution to screen and mitigate these potential adverse effects in individuals with COPD. Future work needs to be undertaken to disseminate and implement findings from this study without creating inconvenience to travelers.
